# Immunomodulatory Effects of *Galleria mellonella* and *Alphitobius diaperinus* Protein Digestates Generated Using a Modified INFOGEST Protocol – *In Vitro* and *In Silico* Evidence

**DOI:** 10.1021/acs.jafc.6c09355

**Published:** 2026-09-25

**Authors:** Zidan Ma, Christine Boesch, Martin Mondor, Oscar Abel Sánchez-Velázquez, Alan Javier Hernández-Álvarez

**Affiliations:** † School of Food Science and Nutrition, 4468 University of Leeds, Leeds LS2 9JT, U.K.; ‡ School of Food Science, Southwest University, Chongqing 400715, China; § National Alternative Protein Innovation Centre (NAPIC), Leeds LS2 9JT, UK; ∥ Department of Chemical Engineering and Biotechnological Engineering, 248219 Université de Sherbrooke, Sherbrooke, Quebec J1K 2R1, Canada; ⊥ Institute of Nutrition and Functional Foods (INAF), Université Laval, Quebec, Quebec G1V 0A6, Canada

**Keywords:** bioactive peptides, inflammation, NF-κB pathway, Nrf2 pathway, edible insects, proinflammatory cytokines

## Abstract

Insect-derived peptides have attracted increasing attention due to their potential health-promoting properties. This study investigated peptides released from *Galleria mellonella* (W) and *Alphitobius diaperinus* (B) proteins following a modified static INFOGEST protocol. Digestion-derived peptides were identified by LC-MS/MS and evaluated for anti-inflammatory activity using *in vitro* assays, bioinformatic prediction, and molecular docking, focusing on IKK-β as a key regulator of the NF-κB pathway. At 500 μg protein/mL, W-I2h and B-I2h reduced NF-κB reporter activity by 28.7% and 53.5%, respectively, and decreased nitric oxide production by 31%. Digestates also reduced IL-6, TNF-α, IFN-γ, and IL-1β secretion at selected concentrations and decreased IL-6 and IL-1β mRNA expression. Conversely, 25 μg protein/mL increased IL-1β secretion by 109.4% and 102.1%, respectively. Increased HO-1 and γGCS transcripts suggested modulation of Nrf2-responsive gene expression, although pathway activation was not confirmed. These findings demonstrate concentration-dependent immunomodulatory effects and identify candidate peptides for future validation.

## Graphical abstract

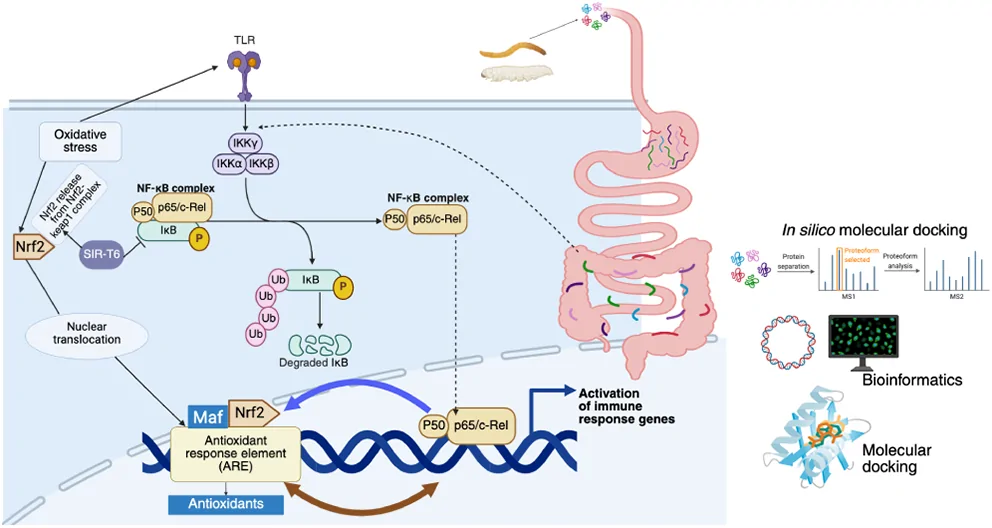

## 1 Introduction

Inflammation is a complex and essential defensive response of the host immune system. In most cases, it is a self-regulated immune process that resolves once the immune challenge is eliminated. However, dysregulated inflammatory responses or a disruption in physiological immune homeostasis can lead to chronic systemic damage.^1^ Nuclear factor (NF)-κB is a key transcription factor that controls the inflammatory response.^1^ The canonical activation of the NF-κB signaling pathway involves the phosphorylation of IκB proteins mediated by IκB kinases (IKKs), which are subsequently ubiquitinated by ubiquitin ligases and degraded by the 26S proteasome. Upon degradation of IκB (i.e., IκBα can be degraded by ubiquitinating enzymes),^2^ the NF-κB dimer dissociates from the NF-κB/IκB complex and translocates to the nucleus, binds to the promoter region on the target gene DNA, thereby initiating the transcription of NF-κB target genes.^3^ Furthermore, the NF-κB pathway is known to crosstalk with the nuclear factor erythroid 2-related factor 2 (Nrf2) signaling pathway, which plays a crucial role in mitigating oxidative stress. Excessive production of reactive oxygen species (ROS) can activate Nrf2, promoting its translocation into the nucleus, where it binds to antioxidant response elements and upregulates the expression of cytoprotective enzymes such as heme oxygenase-1 (HO-1). In turn, Nrf2 activation can suppress ROS-mediated NF-κB signaling, thereby reducing inflammation.^4,5^ Several bioactive compounds, including quercetin, have been shown to downregulate various inflammatory cytokines such as IL-1β while simultaneously inhibiting the NF-κB pathway and enhancing HO-1 expression through the activation of the Nrf2 pathway.^6,7^ Similarly, the monoterpenoid loganin has been reported to mitigate inflammation by acting as a potent ROS scavenger via the Nrf2/HO-1 axis in macrophages.^4^ In addition, polysaccharides from *Christia vespertilion* have been reported to exert selective biological effects by reducing the Bcl2/Bax ratio and inhibiting G1/S cell cycle progression.^7^ However, whether insect protein-derived peptides can activate the oxidative stress-protective Nrf2 pathway, as plant-derived peptides and marine-derived peptides have been extensively documented as Nrf2 activators with antioxidant and anti-inflammatory properties—do, remains unclear, and insect peptides have received considerably less attention in this context.^8^

The demand for food-derived anti-inflammatory peptides arises from their potential to mimic or complement the role of endogenous anti-inflammatory agents. During inflammatory responses, neuropeptides and lipid mediators are produced to regulate inflammation, maintain immune homeostasis, and promote its resolution.^9^ Similarly, food-derived compounds, particularly those with anti-inflammatory properties, can support these natural processes by modulating inflammatory pathways and attenuating excessive immune activation. Incorporating these peptides into the diet offers a promising strategy for managing chronic inflammation and associated diseases through natural dietary interventions.^10^ Bioactive peptides typically contain between 2 and 20 amino acids and have been identified in both animal and plant protein sources, including egg white, fish, bovine α-lactalbumin, and soybean.^11–15^ More recently, other alternative protein sources, such as insects, have gained increasing attention for their potential bioactive properties. While over 2,000 edible insect species have been reported to date,^16,17^ only a limited number of insects have been explored for bioactive peptide production. The main insect species studied so far include silkworm (*Bombyx mori*), mealworm (*Tenebrio molitor*), cricket (*Gryllus bimaculatus*), and locust (*Schistocerca gregaria*).^18^ For instance, the peptide APPPKK from silkworm and KVEGDLK, YETGNGIK, AIGVGAIR, IIAPPER, and FDPFPK from mealworm and cricket have been identified as antihypertensive peptides.^19,20^ The study suggests that insects reared under diverse or challenged conditions may naturally produce a broader and more potent repertoire of stress-related peptides. Consequently, the bioactive peptide profiles derived from insect proteins are likely a direct reflection of the species’ inherent physiological programming and environmental interactions.^21^ Tetrapeptides from house cricket (*Acheta domesticus*) protein hydrolyzates were shown to reduce ROS accumulation by 2-fold and upregulate Nrf2, SOD, CAT, and GPx4 gene expression in H_2_O_2_-exposed HepG2 and SH-SY5Y cells.^22^ Similarly, protein hydrolyzates from black soldier fly (*Hermetia illucens*) suppressed LPS-induced ROS and nitrite production in L-929 cells while upregulating HO-1 and SOD.^23^ Therefore, expanding research into bioactive peptides from a broader range of edible insect species will contribute to a deeper understanding of the potential of insect proteins as a novel and sustainable source of bioactive peptides.^10,24^

We hypothesized that intestinal-phase digestates derived from*Galleria mellonella*and*Alphitobius diaperinus*proteins would attenuate selected NF-κB-associated inflammatory responses and modulate the expression of Nrf2-responsive genes in LPS-stimulated macrophages. The study therefore evaluated the biological effects of the complete <10 kDa digestate fractions generated from a modified static *in vitro* INFOGEST protocol using cellular assays, and applied LC-MS/MS, bioinformatic predictions, and exploratory molecular docking to prioritize candidate peptide sequences for subsequent direct validation. Two insect species,*Galleria mellonella*and*Alphitobius diaperinus*, were selected for the purpose of comparison, and the potency of the digestates (2 h intestinal phase, MW < 10 kDa) to attenuate NF-κB signaling via RAW-Blue cells was investigated. Peptides derived from intestinal digestion were identified, and bioinformatic tools were applied to predict anti-inflammatory potential, which was followed by selection of the most potent peptides for molecular docking with IKK-β as further confirmation of the anti-inflammatory properties. The computational analyses were intended for candidate prioritization and not as confirmation of the anti-inflammatory activity of individual peptides or inhibition of IKK-β. RAW264.7 macrophages were used to confirm the effects of the digestates on the secretion of nitric oxide (NO) and pro-inflammatory cytokines such as IL-6, TNF-α, IFN-γ, and IL-1β, as well as related mRNA expression. Further, mRNA expression of Nrf2 target genes HO-1 and γGCS was investigated. This is the first study to investigate anti-inflammatory peptides derived from digested insect proteins and their effects on the NF-κB and Nrf2 pathways.

It is also the first to identify potential anti-inflammatory peptides from*Galleria mellonella*and*Alphitobius diaperinus* using an *in silico* approach. Previous work from our group characterized the protein quality, structural properties, peptide profiles, transepithelial transport, and antioxidant potential of peptides derived from*Galleria mellonella*and*Alphitobius diaperinus*. The present study extends this work by focusing specifically on anti-inflammatory activity in LPS-stimulated macrophage models, including NF-κB reporter activity, NO production, cytokine secretion, inflammatory gene expression, Nrf2-associated target gene expression, and *in silico* prioritization of candidate peptides. Therefore, the novelty of this study lies in connecting insect-protein gastrointestinal digestates with macrophage inflammatory signaling and identifying candidate peptides for future mechanistic validation

## 2 Materials and Methods

### 2.1 Materials

Live waxworms (W; *Galleria mellonella*) were purchased from a commercial supplier (Livefood4u, Sheffield, UK) and frozen,^25^ while frozen buffalo worms (B; *Alphitobius diaperinus*) were purchased from the company Kiezebrink (Kiezebrink Ltd., Church Farm, Suffolk, UK). Both insects were freeze-dried, and protein was extracted by alkaline solubilization followed by isoelectric precipitation and freeze-drying, according to Ma et al.^26^ Total nitrogen content was determined using the Dumas method (vario MACRO cube, Langenselbold, Germany). For samples with low protein content, rice flour (N = 1.35 ± 0.04) was used as the standard, while EDTA (N = 9.58 ± 0.04) served as the standard for samples with high protein content.^27^ The protein content of waxworm and buffalo worms protein concentrates were 87.61% and 71.55%, respectively.

### 2.2 *In Vitro* Intestinal Digestion (INFOGEST)

The *in vitro* digestion of the protein concentrates was performed according to the static INFOGEST protocol with minor modifications.^24,28^ In brief, 2 g of protein concentrate were mixed with Milli-Q water to make a paste, followed by addition of simulated salivary fluid (SSF), CaCl_2_, amylase (75 IU/mL), and water, and incubated for 2 min at 37 °C. The oral phase was followed by addition of the gastric phase fluid (SGF), pepsin (200 U/mL), CaCl_2_, and water, then incubated at 37 °C for 2 h. Afterwards, intestinal phase fluid (SIF), an intestinal enzyme mix, CaCl_2_, and water were added to the suspension and incubated at 37 °C for 2 h. Because the study focused specifically on proteolysis, bile salts, pancreatic lipase, and co-lipase were omitted from the intestinal phase. Accordingly, this model does not reproduce lipid digestion or bile-mediated solubilization. Furthermore, the starting protein concentrates exhibited a high protein content and reduced fat content (*Alphitobius diaperinus*- 71.55% protein and 2.71% fat; *Galleria mellonella*- 87.61% protein and 4.52% fat). The digestates were heated in 70 °C water for 10 min to stop enzymatic reactions and subsequently centrifuged at 4,500 g for 15 min at 4 °C. The supernatants were further processed via spin column (Pierce^TM^ Protein Concentrator PES, Thermo Fisher Scientific, USA) to separate and enrich low MW proteins from the mixture, with a 10 kDa cut off. The soluble protein content of the fractions below 10 kDa was determined by BCA assay and kept for further analysis.

#### 2.2.1 Amino Acid Profiles

The digested insect fractions (W-I2h and B-I2h) below 10 kDa were freeze-dried and 2 mg of each were hydrolyzed in 6 N HCl (4 mL) at 110 °C for 24 h in tubes sealed under nitrogen.^29^ In brief, after HCl hydrolysis, amino acids were determined via HPLC (Agilent Technologies, Inc., Wilmington, DE, USA). To this end, amino acids were derivatized with diethyl ethoxymethylenemalonate, followed by injection (20 μL) and separation using a 300 mm × 3.9 mm i.d. reversed-phase column. The isocratic elution system contained 25 mM sodium acetate, 0.02% sodium azide (pH 6)/acetonitrile (91:9, v/v) with a flow rate of 0.9 mL/min (Novapack C18, 4 μm; Waters, Milford, MA, USA). The tryptophan content was determined by HPLC after basic hydrolysis according to Yust et al.^30^

#### 2.2.2 Peptide Identification

The peptides collected from the I2h fraction were analyzed by DDA-PASEF and LC-MS/MS according to Ma et al.^24^ Briefly, a 2-μg aliquot of each sample was loaded onto EvoTip One tips for desalting and HPLC introduction. Peptides were separated over a 60SPD gradient using an EvoSep One UPLC. Data were acquired using a Bruker timsTOF HT mass spectrometer. For database searching, MS data in “.d” format were processed and searched using FragPipe (v21.1), with nonspecific enzymatic cleavage. All matched peptides were filtered to a 1% FDR and a minimum probability of 0.99.

### 2.3 Bioactivity Prediction Using Bioinformatics

LC–MS/MS-identified peptide sequences from W-I2h and B-I2h were initially screened using PeptideRanker (http://distilldeep.ucd.ie/PeptideRanker/). Sequences meeting the predefined PeptideRanker threshold of >0.60 were subsequently evaluated using PreAIP (http://kurata14.bio.kyutech.ac.jp/PreAIP/index.php), for which a score ≥0.468 was considered indicative of predicted anti-inflammatory potential. The two highest-scoring eligible peptides from each insect species were taken forward, resulting in four candidate peptides in total. Predicted toxicity and allergenicity were assessed using ToxinPred (https://webs.iiitd.edu.in/raghava/toxinpred/multi_submit.php/) and AllergenFP (https://ddg-pharmfac.net/AllergenFP/feedback.py), respectively. This computational workflow was used to prioritize candidate sequences and does not establish their biological activity. The structure of the selected peptides was analyzed by http://pepcalc.com/.

### 2.4 Targeted Peptides’ Molecular Docking with the IKK-β Active Site

The four candidate peptides selected using the bioinformatic workflow described in Section 2.3 were subjected to exploratory molecular docking against the predefined IKK-β binding region according to Yang et al.^31^ Docking was used to generate hypotheses regarding geometrically plausible peptide–protein interactions and was not considered confirmation of binding affinity, target engagement, IKK-β inhibition, or anti-inflammatory activity. The software platform Discovery Studio (BIOVIA Discovery Studio Visualizer, San Diego, USA) was applied for molecular docking. The IKK-β crystal structure (PDB:3RZF) was retrieved from the Protein Data Bank (PDB), and the selected peptides’ 3D structures were generated from ChemDraw 22.0.22 (PerkinElmer, Waltham, USA) with the energy minimized (set to the lowest-energy state). Before docking, the IKK-β crystal structure was conducted to remove water and the native ligand, and polar hydrogens and charges were added to the structure. Then the docking site was set as the IKK-β binding region, X: 89, Y: −26, and Z: 57; radius: 15 Å. The 3D interactions were visualized with the software PyMOL (PyMOL Molecular Graphics System, Schrödinger, LLC).

### 2.5 Cell Culture

#### 2.5.1 Cultivation of RAW-Blue Cells and RAW264.7 Macrophages

RAW264.7 macrophage cells (TIB-71^TM^ purchased from the European Collection of Authenticated Cells, Salisbury, UK) and RAW-Blue cells (obtained from InvivoGen, Toulouse, France) were cultivated in Dulbecco’s Modified Eagle Medium (DMEM) (Gibco, GlutaMAX^TM^) containing 4.5 g/L D-glucose and 110 mg/L pyruvate, with 10% FBS and 1% penicillin-streptomycin. Both cell lines were grown under standard conditions at 37 °C, in a 5% CO_2_ humidified atmosphere, and passaged at around 80% confluence.

#### 2.5.2 Cell Viability

The viability of RAW264.7 macrophages and RAW-Blue cells was determined using a neutral red assay according to Repetto et al.^32^ In brief, both macrophage lines were seeded at 5 × 10^4^ per well and grown until confluence reached around 80%. Then, the medium was replaced with medium containing W-I2h and B-I2h at different concentrations of 250, 500, 1,000, 2,000, and 2,500 μg of soluble protein/mL and incubated for 24 h. Subsequently, the digestate-containing medium was removed and replaced with DMEM medium with neutral red (40 μg/mL). The cells were then washed by DPBS before adding 200 μL of bleach solution per well. The plate was incubated for 10 min at 37 °C with shaking and read at 540 nm (SPARK 10M, Multimode Microplate Reader, Tecan, Switzerland).

### 2.6 *NF**-*** κB Reporter Assay

RAW-Blue cells grown to 80% in 24-well plates were incubated with W-I2h and B-I2h (25, 50, 100, 250, and 500 μg protein/mL) for 1 h, followed by application of lipopolysaccharide (LPS) as an inflammatory stimulator for 24 h.^33^ The medium was removed, mixed with QUANTI-Blue^TM^ substrate (1:4, v/v), and incubated for 1 h at 37 °C before the absorbance was read at 620 nm using a SPARK 10M plate reader. Cells without LPS treatment and without digestate were used as negative controls, and the cells with LPS treatment and no digestate were used as positive controls.

### 2.7 Inflammation Cytokine Secretion Assay

The secretion of cytokines TNF-α, IL-6, IL-1β, and IFN-γ was determined by ELISA according to the manufacturers’ protocols (DuoSet ® ELISA, Biotechne R&D systems, Abingdon, UK). They were selected as representative proinflammatory cytokines as they are key downstream products of NFκB activation in LPS-stimulated macrophages. Briefly, 80% confluent RAW264.7 macrophages (in 24-well plates) were incubated with W-I2h and B-I2h at soluble protein concentrations of 25, 100, 250, and 500 μg protein/mL. After 1 h, 1 μg/mL of LPS was added to stimulate cells for 24 h, after which time the medium was collected and frozen at −80 °C.^34^ The NO assay was performed according to Schmölz, et al.^35^ After 24 h, 100 μL of cell medium was mixed with 100 μL of Griess reagent and incubated for 10 min at room temperature. The absorbance was measured at 550 nm with the plate reader. The positive control was DMEM with 1 μg/mL LPS, and the negative control was DMEM only without LPS stimulation.

### 2.8 Real-Time Quantitative PCR

RAW264.7 macrophages, 80% confluent (in 12-well plates), were incubated with medium containing W-I2h and B-I2h (50, 100, 250, and 500 μg of soluble protein/mL) for 1 h, followed by LPS (1 μg/mL) addition. After 6 h of incubation, the medium was removed, the cells were washed using DPBS, and harvested for subsequent RNA isolation (PureLink® RNA Mini Kit, Life Technologies, USA). The RNA concentration and purity were determined by spectrophotometry. For each sample, 0.5 μg of RNA was reverse transcribed into cDNA with the iScript cDNA synthesis kit (Bio-Rad, Watford, UK). Real-time qPCR was performed using iTaq Universal SYBR Green reagent (Bio-Rad, Watford, UK) and run on a StepOne^TM^ Real-time PCR system (Thermo Fisher Scientific, Loughborough, UK), including melt-curve analysis to confirm the targeted amplicon size. The primer sequence details for the target genes have been reported previously,^36^ with β-actin used as the housekeeping gene to normalize target gene expression, and relative gene expression was calculated according to Livak and Schmittgen^37^

### 2.9 Statistical Analysis

Protein concentrate obtained from one source material batch per insect species was subjected to three independently initiated INFOGEST digestion runs. Each independently generated digestate was evaluated in a separate cell culture experiment using a separate cell passage, with three technical wells per condition. Technical replicates were averaged before statistical analysis, and the independently generated digestion/cell experiment was treated as the experimental unit (n = 3). Accordingly, the study characterizes variability between digestion and cell culture experiments but does not assess variability between different source materials or production batches. Thus, all the experiments were carried out in triplicate using the same batch of digestates digested in triplicate, and data are presented as the mean ± SD of three independent experiments. Statistical analysis was performed using OriginPro 2021 (Version 9.8.0.200 OriginLab Corporation, Northampton, MA, USA). Data were analyzed by one-way ANOVA, followed by Tukey’s post hoc test. Significant differences between groups were determined using Tukey’s test, with a significance threshold of *p* < 0.05. All graphical representations were plotted using OriginPro 2021.

## 3 Results and Discussion

### 3.1 INFOGEST Digestion

#### 3.1.1 The Amino Acid Profile of the Soluble Digestates

Table 1 presents the amino acid profiles of the soluble fractions (MW < 10 kDa) of W-I2h and B-I2h. Soluble digestates represent the fraction of proteins that remain bioaccessible after gastrointestinal digestion. For W-I2h, in general, the three most abundant essential amino acids following digestion were Lys, Phe and Leu. This is in agreement with the results reported by Poelaert et al.,^38^ who found that intestinal digestates of*Tenebrio molitor* were also rich in Lys and Leu, with concentrations of 10.0 and 10.2 g/100 g protein, respectively. Similarly,*Acheta domesticus* *in vitro* digestates contained 9.3 g/100 g Lys and 8.9 g/100 g Leu. However, W-I2h exhibited higher Lys and lower Leu concentrations compared to*Tenebrio molitor* pupae intestinal digestates, with values of 13.5 and 6.3 g amino acids/100 g protein, respectively.^39^ Similarly, for B-I2h, the intestinal digestates were also rich in Lys, Phe and Leu, with concentrations of 18.0, 12.4 and 5.6 g amino acids/100 g protein, respectively. Notably, the Phe content in B-I2h (12.4 g amino acids/100 g protein) was significantly higher than that observed in W-I2h (6.9 g amino acids/100 g protein), but lower than that of whey protein digestates (∼15 g amino acids/100 g protein). Furthermore, the total essential amino acid content of B-I2h (44.9 g amino acids/100 g protein) was higher than that observed for W-I2h (39.8 g amino acids/100 g protein).

**Table 1:** Total Amino Acid Profile for *Galleria mellonella* (W) Worm and *Alphitobius diaperinus* (B) Worm Intestinal Digestates (<10 kDa)[Table-fn tbl1fn1]

	**Asp**	**Ser**	**Glu**	**His**	**Gly**	**Thr**	**Arg**	**Ala**	**Pro**	**Tyr**	**Val**	**Met**	**Cys**	**Ile**	**Trp**	**Leu**	**Phe**	**Lys**
W-I2h	1.23 ± 0.03^a^	1.36 ± 0.05^a^	7.82 ± 0.3^a^	2.58 ± 0.13^a^	2.40 ± 0.05^a^	1.38 ± 0.00^a^	20.96 ± 0.44^a^	4.73 ± 0.11^a^	5.00 ± 1.19^a^	10.28± 0.43^a^	3.78 ± 0.02^a^	0.33 ± 0.03^a^	0.30 ± 0.12^a^	3.82± 0.23^a^	1.15 ± 0.06^a^	6.28 ± 0.63^a^	6.89 ± 0.22^a^	13.54 ± 0.31^a^
B-I2h	0.34 ± 0.01^b^	0.34 ± 0.19^b^	2.11 ± 0.14^b^	3.13 ± 0.03^b^	2.42 ± 0.07^a^	0.59 ± 0.03^b^	17.62 ± 0.71^b^	2.89 ± 0.14^b^	3.74 ± 0.09^b^	19.67 ± 0.35^b^	2.83 ± 0.80^b^	0.36 ± 0.23^a^	0.36 ± 0.01^a^	1.37 ± 0.20^b^	1.07 ± 0.02^b^	5.56 ± 0.04^b^	12.42 ± 0.21^b^	17.95 ± 0.13^b^

i Data are expressed as mean ± SD, p < 0.05, Tukey test. Different letters indicate statistical difference between Galleria mellonella (W) worm and Alphitobius diaperinus (B) worm intestinal digestates (I2h) (< 10 kDa). Note: The content of each amino acid is expressed in g amino acid/100 g protein in the freeze-dried soluble digestates.

Other than the rich amino acids, the hydrophobic amino acid content in W-I2h is 34.1%, and that in B-I2h is 31.9%. The relatively high hydrophobic amino acid content of W-I2h and B-I2h may contribute to peptide interactions with cell membranes or molecular targets involved in inflammatory signaling. However, amino acid composition alone does not confirm anti-inflammatory activity and should be interpreted alongside cellular assays and peptide-level validation. The omission of bile salts and lipolytic components may affect peptide release, solubility, micellar interactions, and bioavailability; therefore, the findings should be interpreted as evidence from protein-focused intestinal digestates rather than as a complete prediction of *in vivo* intestinal exposure.

### 3.2 Selected Peptides Docking with IKK-β

As highlighted by the European Food Safety Authority (EFSA), bioinformatics is a powerful tool for predicting the biological properties of peptides.^40^ In this study, molecular docking, a computational prediction method, was employed to predict the binding interactions between selected peptides (ligands) and IKK-β, to demonstrate that the peptides (with the highest anti-inflammatory scores) have the potential to bind with IKK-β to exert their anti-inflammatory function. IKK-β is the predominant catalytic subunit of the IκB kinase (IKK) family and plays a critical role in activating the NF-κB signaling pathway. More specifically, IKK-β facilitates the phosphorylation and subsequent degradation of IκB, leading to the detachment of IκB from the NF-κB complex and the release of the active NF-κB subunit. Given its central role in NF-κB activation, IKK-β was selected as the target for investigating the inhibitory effects of the selected peptides.^41^

For waxworm intestinal digestate, two peptides with the highest predicted anti-inflammatory scores were identified: GGSAASLGDHPHLGGLL (0.615) and FGPISIGNPPQSF (0.546). Similarly, for B, two peptides were selected: PEQALDPINKPTFW (0.518) and DAFPEQALDPINKPTF (0.526). Mudgil et al.^42^ showed that peptides with strong anti-inflammatory effects often contain amino acids like arginine, leucine, and lysine. The presence of arginine or lysine at the start (N-terminal) or end (C-terminal) of a peptide can enhance its anti-inflammatory effects, whereas hydrophobic amino acids present in peptides, including alanine, glycine, proline, valine, isoleucine, leucine, methionine, phenylalanine, tyrosine, and tryptophan, contribute to antioxidant capacity, and has been linked as well to anti-inflammatory activity enhancement.^43^ Although the selected peptides do not contain R or K at the N-terminal or C-terminal, GGSAASLGDHPHLGGLL (0.615) demonstrated a higher anti-inflammatory score compared to the peptide with K at the terminal, VSGVFSSFMTPEKRMIK (0.57), from bird nest and the known IKK-β inhibition peptide GEGGPQGPR (0.52).^44^ Mechanistically, a study recently demonstrated that tiger nut-derived antioxidant peptides act through a dual mechanism: direct radical scavenging via electron transfer from aromatic/hydrophobic residues, and indirect activation of the Nrf2 pathway through binding to Keap1 and disruption of the Keap1Nrf2 interaction.^45^ The high abundance of hydrophobic residues (L, P, G, A, and H) in the peptide GGSAASLGDHPHLGGLL identified in this study is consistent with the structural features associated with Keap1 binding and radical stabilization. Thus, its high predicted antiinflammatory activity may reflect both direct ROS scavenging capacity and Nrf2-mediated upregulation of endogenous defenses.

Molecular docking was employed to evaluate the inhibitory potential of the selected peptides against IKK-β. Previous studies have identified key IKK-β binding sites, including Ala42, Asp166, Arg31, Cys99, Glu97, Glu100, Glu149, Gly102, Ile165, Lys106, Lys147, Met96, Thr23, Tyr98, Val29, and Val152.^31,46,47^ The peptides selected in this study exhibited interactions with several of these critical residues (Figure 1). Specifically, peptide FGPISIGNPPQSF (peptide 1) formed hydrogen bonds with Arg31, Glu100, Glu149, and Gly102, while PEQALDPINKPTFW (peptide 3) interacted with Tyr98, Lys106, Glu149, and Lys147. DAFPEQALDPINKPTF (peptide 2) and GGSAASLGDHPHLGGLL (peptide 4) exhibited additional unique binding interactions. Peptide 2 bound to Asn109, Asp103, Arg20, Tyr28, Thr23, and Thr185, while peptide 4 interacted with Asn109, Arg105, Asp103, Tyr188, Glu214, and Gly218. These findings suggest that the selected peptides exhibit binding affinity for IKK-β, potentially interfering with NF-κB activation and thereby contributing to their anti-inflammatory properties. To place the docking results of the present study within experimentally validated structure-activity relationships, it is noteworthy that a recent study^48^ reported that the reduction in peptide bioactivity after simulated intestinal digestion was correlated with a decrease in proline content, a feature also observed in the current selected peptides.

**Figure 1:**
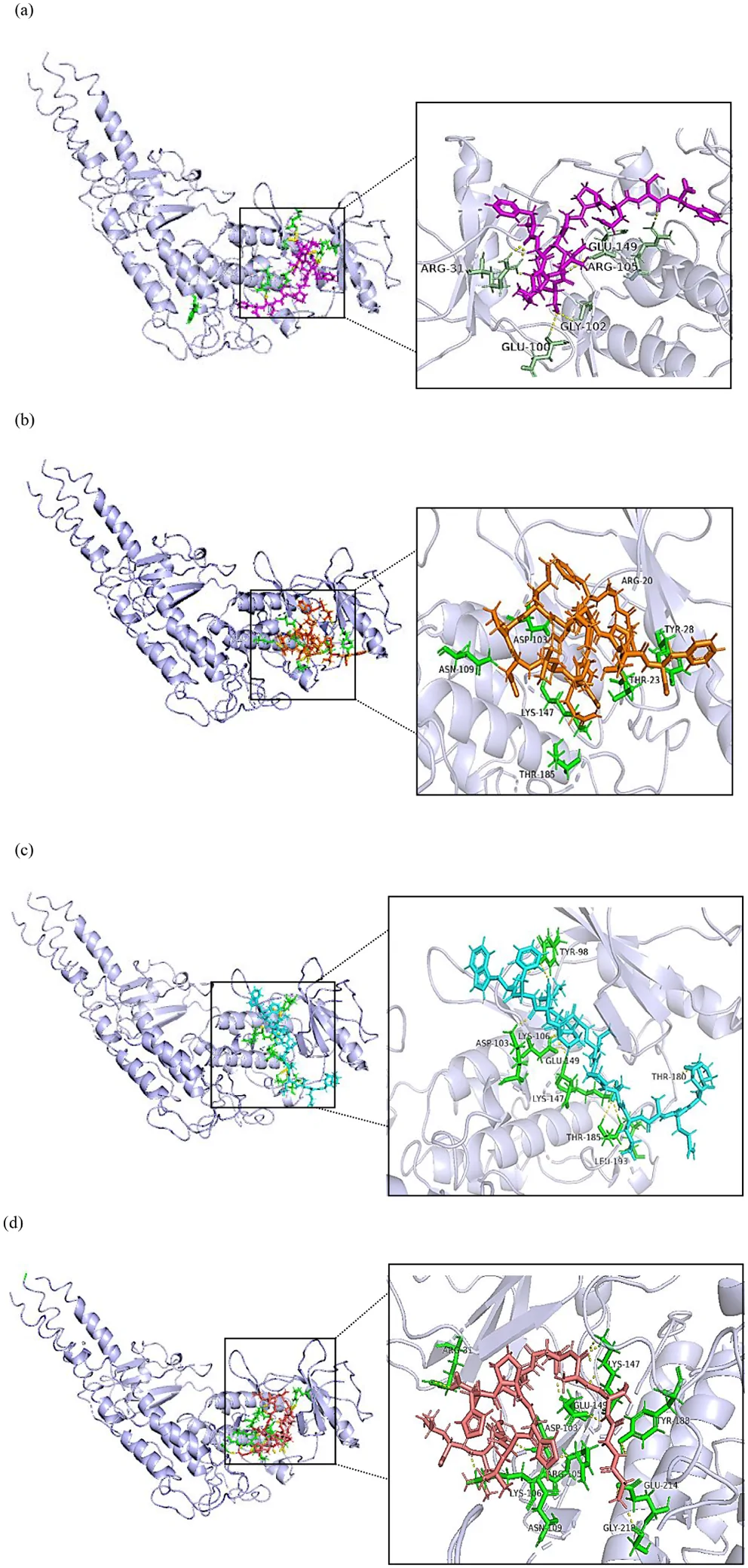
3D and 2D docking diagrams of (a) FGPISIGNPPQSF, (b) DAFPEQALDPINKPTF, (c) PEQALDPINKPTFW, and (d) GGSAASLGDHPHLGGLL with the active site of IKK-β.

### 3.3 Structure Analysis of the Selected Anti-Inflammatory Peptide

The structure analysis of the selected peptides is shown in Table 2. It has been reported that several factors, including molecular weight, length of the peptide chain, polar amino acids, and aromatic amino acids, play important roles in anti-inflammatory activity. However, beyond anti-inflammatory potential, peptide stability during gastrointestinal digestion is essential for *in vivo* functionality. Tong et al.^49^ recently showed that structural complexation (e.g., V-type crystalline formation) can enhance resistance to digestion by limiting enzyme accessibility. By extension, specific structural attributes of the peptides selected in this study, including chain length, hydrophobicity, or propensity to adopt secondary structure, may similarly influence their resistance to proteolysis. Similar to the anti-inflammatory peptide analysis reported by Rivera-Jiménez et al.,^50^ in the current study, the MWs of most of the anti-inflammatory peptides are around 1,000 Da and a length between 4 and 20 amino acids. The MW and length of the four selected peptides are similar to the range of the reported anti-inflammatory peptides.

**Table 2:** Structure Analysis of the Selected Anti-Inflammatory Peptides^a^

Peptide sequence	M_w_(Da)	pI	Net charge (pH7)	Water solubility	PCL	Polar AA (%)	Aromatic AA (%)	P (%)	G (%)	Q (%)
FGPISIGNPPQSF	1360.51	3.45	0	poor	13	31	15	23	15	8
DAFPEQALDPINKPTF	1802.98	3.54	–2	good	16	19	19	19	0	6
PEQALDPINKPTFW	1655.85	3.93	–1	good	14	21	14	21	0	7
GGSAASLGDHPHLGGLL	1558.69	6.04	–0.8	poor	17	12	0	6	29	0

a *M*_w_, molecular weight; pI, isoelectric point; PCL, peptide chain length; AA, amino acids; P, presence of a proline residue; G, presence of a glycine residue; Q, presence of a glutamine residue.

Generally, peptides rich in proline, glycine, or glutamine have great potential against inflammatory responses. The tripeptides IPP and VPP derived from milk, both recognized as potent anti-inflammatory agents, are rich in proline (P).^51^ The selected peptides in this study contain a high percentage of P, approximately 20%, except for GGSAASLGDHPHLGGLL. In addition, the presence of proline has been related to a lower degree of gastrointestinal digestion and, therefore, higher bioaccessibility and more likely absorption upon oral consumption, a factor that can be beneficial for future therapeutic applications.

Further, it has been shown that anti-inflammatory peptides containing aromatic amino acids (10–50%) are less susceptible to intestinal peptidases and proteases,^50^ which is similar to the values reported in this study. However, GGSAASLGDHPHLGGLL does not contain aromatic amino acids but shows high anti-inflammatory properties, which can be linked to the high glycine content (G), and reference to the soybean anti-inflammatory peptide YGGGGE, composed of 67% glycine.^52^ On the other hand, the net charges of the selected peptides are negative and zero, which is in line with the anti-inflammatory peptides DAPAPPSQLEHIRAA and AADGPMKGILGY from*Lateolabrax maculatus*, which have net charges of −0.9 and 0, respectively.^53^ Similarly, the anti-inflammatory peptide DQWL from whey protein showed a net charge of −1.^54^

On the other hand, no obvious relationship has been found between peptide solubility and potential bioactivity.^50^ Among the selected anti-inflammatory peptides, two exhibited good water solubility, while the other two were poorly water-soluble (Table 2). This observation is consistent with the findings reported by Rivera-Jiménez et al.^50^ that no obvious relationship has been found between the solubility of the peptides and their potential bioactivity.

### 3.4 Inhibition of NF-B Activation in LPS-Stimulated RAW-Blue Cells

Prior to evaluating the anti-inflammatory activity of peptides in RAW264.7 macrophages, the cytotoxicity of intestinal digestates (MW < 10 kDa) generated from gastrointestinal digestion was assessed via the Neutral Red assay. As shown in Figure 3A, at concentrations ≤500 μg/mL, neither W-I2h nor B-I2h exhibited any significant impact on cell viability. However, at concentrations of 1,000 μg/mL and above, cell viability was significantly decreased for both W-I2h and B-I2h, dropping below 70%. Neither W-I2h nor B-I2h promoted the proliferation of RAW264.7 macrophages, which is the same as the findings of Lee et al.,^55^ who reported that*Protaetia brevitarsis*proteins at a maximum concentration of 100 μg/mL also did not reduce cell viability. Based on these results, for both W-I2h and B-I2h, a concentration of 500 μg/mL was selected as the nontoxic concentration for further experiments.

To investigate the effects of peptides generated during digestion on inflammatory signaling, the inhibition potential toward NF-κB pathway activation was evaluated using W-I2h and B-I2h samples in RAW-Blue cells (Figure 2). RAW-Blue cells are derived from RAW264.7 macrophages and are specifically designed to monitor activation of the NF-κB signaling pathway via the NF-κB-inducible secreted embryonic alkaline phosphatase (SEAP) reporter gene, which can be read out using a colorimetric assay, as previously reported.^33,56^ The NF-κB activity of the digestates was expressed as the % inhibition relative to LPS-treated control cells (CTL^+^), which was set at 100%. Both W-I2h and B-I2h demonstrated a concentration-dependent effect to inhibit SEAP secretion, although B-I2h demonstrated higher potency at a concentration of 500 μg/mL, with 53.5% inhibition compared to 28.7% in the case of W-I2h. For comparison, Han et al.^33^ reported a 52.8% inhibition of SEAP secretion with casein pepsin hydrolyzates (3–10 kDa, 1,000 μg/mL), which is comparable to B-I2h.

**Figure 2:**
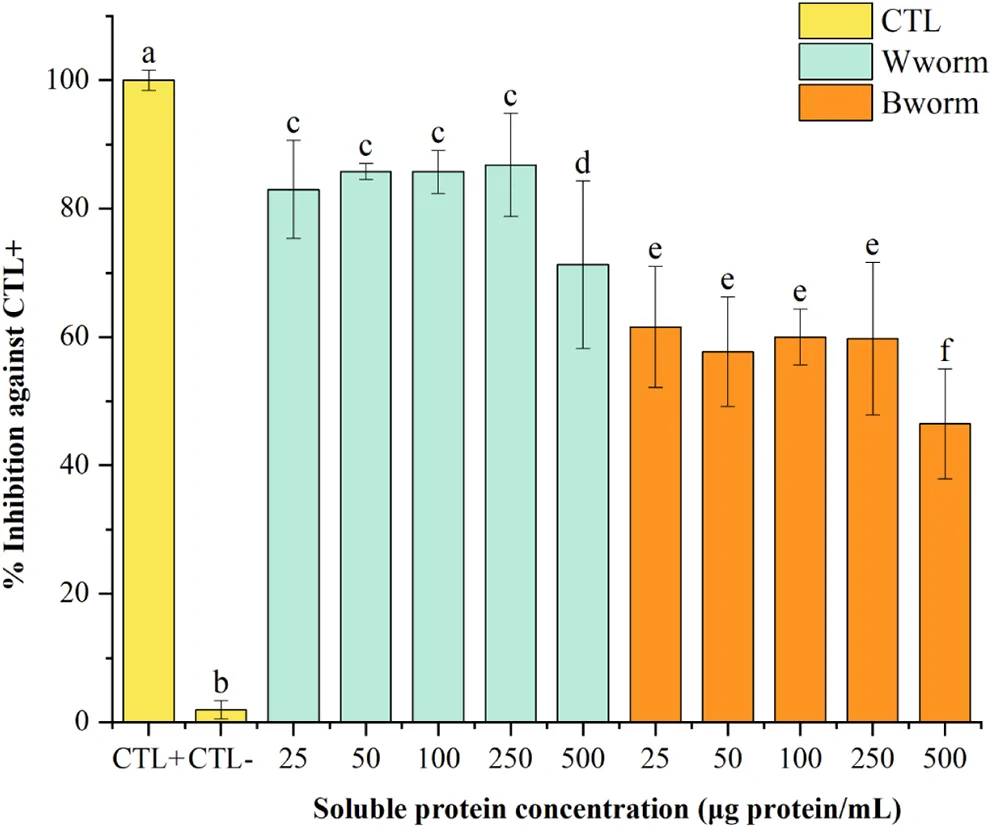
*Galleria mellonella* (W) worm and *Alphitobius diaperinus* (B) worm intestinal digestates (<10 kDa) inhibition effects on the NF-κB response in RAW-Blue cells. Data are the mean with SD of three independent experiments in triplicate. Different letters indicate significant differences, p < 0.05, Tukey test.

### 3.5 Digestate Modulation of Cytokine Secretion and Nitric Oxide Formation

The NO production by RAW264.7 macrophage cells was assessed using the Griess assay in Figure 3B.^35^ As part of their cytotoxic function against pathogens, macrophages produce NO to enhance phagocytic activity.^57^ Excessive NO production contributes to oxidative stress, as it readily reacts with superoxide to form peroxynitrite, a potent oxidant associated with various inflammatory conditions and other health disorders.^58^ Samples containing peptides from the I2h (<10 kDa) were selected for the NO inhibition assay. As shown in Figure 3B, both W-I2h and B-I2h (<10 kDa) reduced NO production in LPS-stimulated macrophages. More specifically, higher concentrations resulted in greater NO reduction, with no significant difference between W-I2h and B-I2h at 500 μg/mL. However, at lower concentrations (25 and 50 μg/mL), B-I2h demonstrated a higher potential for NO suppression. Specifically, B-I2h at 25 and 50 μg protein/mL showed a significant difference compared with W-I2h at the corresponding concentrations. B-I2h inhibited NO production by 13.2% and 15.8%, respectively, whereas W-I2h did not inhibit NO production at either 25 or 50 μg protein/mL.

**Figure 3:**
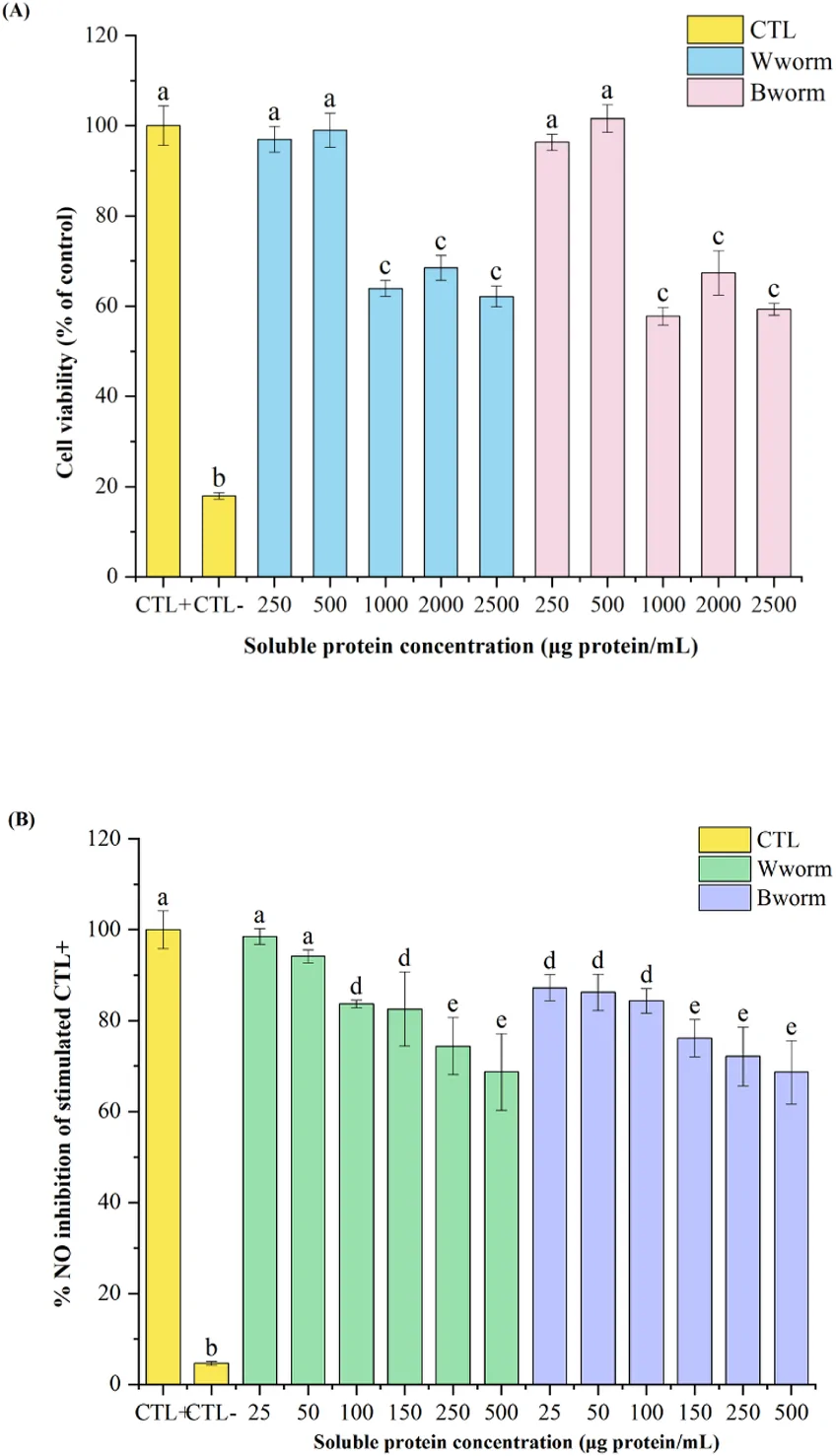
(A) Effects of*Galleria mellonella* (W) worm and*Alphitobius diaperinus*­(B) worm intestinal digestates (<10 kDa) on cell viability in RAW264.7 macrophages. (B) The % nitrite oxide (NO) inhibition of stimulated CTL+ by W-I2h and B-I2h (< 10 kDa). Data are the mean with SD of three independent experiments in triplicate. Different letters indicate significant differences, p < 0.05, Tukey test.

As shown in Figure 4, the effects of W-I2h and B-I2h (< 10 kDa) on the secretion of IL-6, TNF-α, IFN-γ, and IL-1β by RAW264.7 macrophages were evaluated following 24 h of LPS stimulation. These results were compared with those of macrophages treated with LPS alone (CTL+). Among the four cytokines analyzed, IL-6 exhibited the highest secretion levels, which can be explained by the fact that IL-6 is regulated by multiple stimuli; it is not only induced by LPS, but also by cytokines such as TNF-α and IL-1β.^59^

As shown in Figure 4A, both W-I2h and B-I2h (< 10 kDa) exhibited a dose-dependent inhibitory effect on IL-6 secretion. At a concentration of 500 μg protein/mL, W-I2h and B-I2h similarly reduced IL-6 secretion to 3,648 and 3,577 pg/mL, respectively, compared to 4,589 pg/mL in CTL+. This corresponds to inhibition rates of 20.5% for W-I2h and 22.1% for B-I2h.

Interleukin-1β (Figure 4B), a member of the IL-1 cytokine family, is a key mediator of the inflammatory response.^60^ For W, only W-I2h at 500 μg protein/mL significantly inhibited IL-1β secretion compared to CTL+ (27.6% reduction). B-I2h, at 250 and 500 μg protein/mL, showed significant inhibition compared to CTL+, with reductions of 18.8% and 22.8%, respectively. Compared to β-casein, where high doses of β-casein digestates resulted in significant inhibition of both IL-6 (∼31%) and IL-1β (∼50%), both W-I2h and B-I2h were less effective.^61^ At 25 μg soluble protein/mL, W-I2h and B-I2h increased IL-1β secretion by 109.4% and 102.1% (pro-inflammatory effects), respectively, relative to the LPS-treated control. This non-monotonic response demonstrates that the digestates did not exert uniformly anti-inflammatory effects across the tested concentration range. Because complex digestate fractions were evaluated, the mechanism underlying the low-concentration response cannot be attributed to an individual peptide or ascribed to hormesis, Nrf2 signaling, IκBα stabilization, or receptor-specific effects without additional experimental evidence. Further fractionation, direct testing of individual peptides, and assessment of potential non-peptide contributors will be required to explain this response. It has been reported that low concentrations of certain compounds can activate immune cells and stimulate the release of pro-inflammatory cytokines.^62^ This biphasic pattern, characterized by proinflammatory effects at low doses and antiinflammatory effects at higher doses, may be explained by a hormesis-like response. At low concentrations, these compounds may act as mild immunogenic stressors that transiently activate NFκB, whereas higher concentrations engage opposing regulatory pathways, such as Nrf2/HO1 or IκBα stabilization. Additionally, differential receptor occupancy, with partial activation at low doses and antagonism or saturation at higher doses, may contribute to the dose-dependent reversal of IL-1β secretion.

For TNF-α and IFN-γ (Figure 4C and 4D), neither W-I2h nor B-I2h exhibited a clear dose-dependent inhibition from 25 μg protein/mL to 500 μg protein/mL, although significant reduction was observed at all tested concentrations. B-I2h demonstrated a stronger inhibitory effect on TNF-α secretion compared to W-I2h, whereas no significant difference was observed between W-I2h and B-I2h in terms of IFN-γ inhibition. Both W-I2h and B-I2h effectively suppressed IFN-γ secretion, reducing cytokine levels from 93.7 pg/mL to around 24 pg/mL, corresponding to a reduction of approximately 74%.

**Figure 4:**
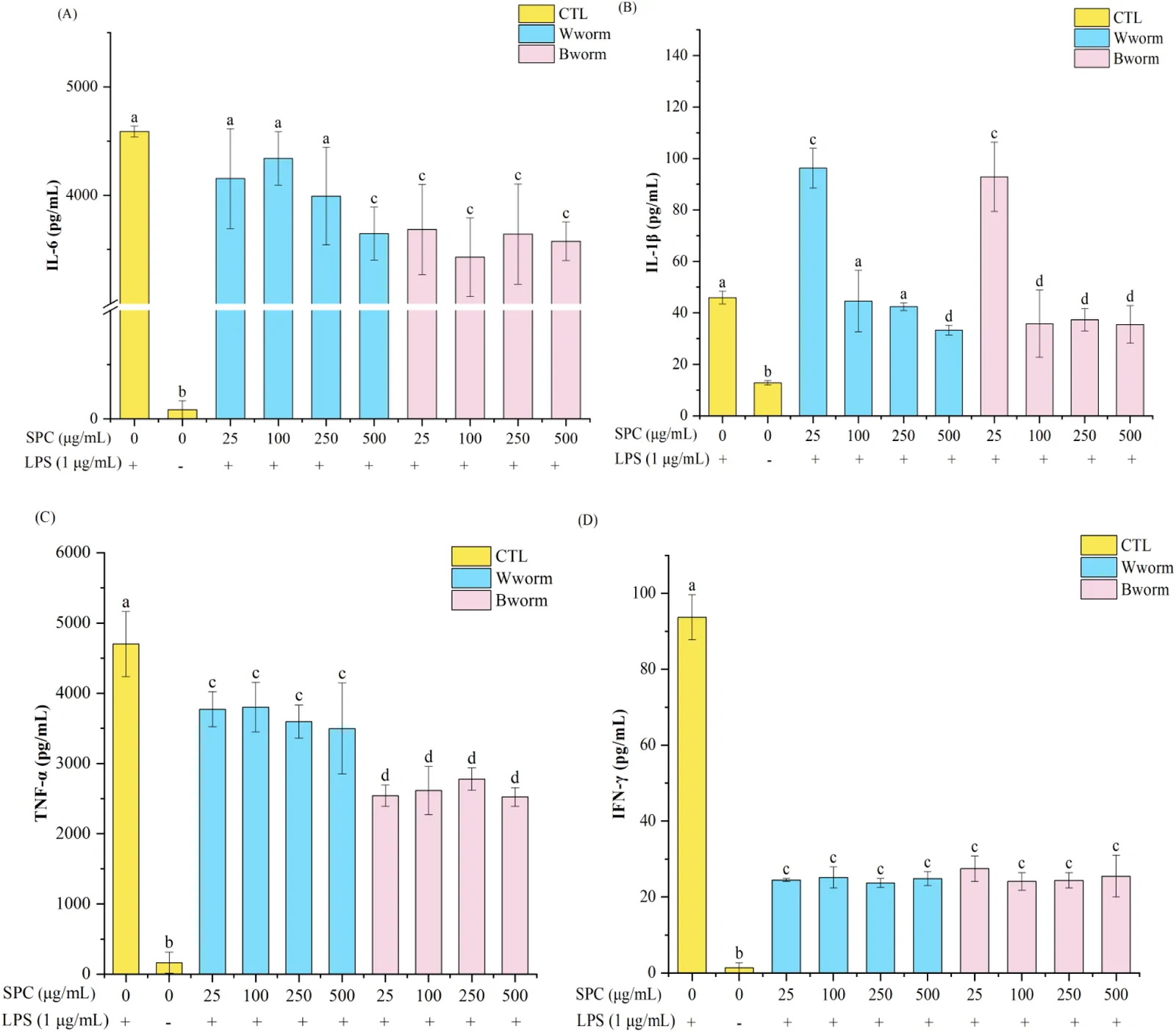
Effects of*Galleria mellonella* (W) worm and*Alphitobius diaperinus*­(B) worm intestinal digestates (< 10 kDa) with different soluble protein concentrations (SPC) on the cytokine release (A) IL-6, (B) IL-1β, (C) TNF-α, and (D) IFN-γ by RAW264.7 macrophages after 24 h LPS treatment. Data are the mean with SD of three independent experiments in triplicate. Different letters indicate significant differences, p < 0.05, Tukey test.

### 3.6 Digestates Modulate the Expression of Pro-Inflammatory Target Genes

To confirm modulation of cytokine secretion at the transcriptional level, the mRNA expression levels of selected target genes were determined. As shown in Figure 5, the LPS-induced upregulation of IL-6 was significantly reduced by W-I2h at all tested concentrations (50–500 μg protein/mL). At 500 μg protein/mL, B-I2h exhibited a similar inhibitory effect on IL-6 expression (65% inhibition) compared to W-I2h (55% inhibition), albeit not at lower concentrations. Both W-I2h and B-I2h at 500 μg protein/mL resulted in a reduction of IL-1β mRNA expression, with W-I2h showing a 51% reduction and B-I2h a 31% reduction. Similar results have been reported for β-casein, where high doses of β-casein digestates resulted in substantial inhibition of both IL-6 (30% inhibition) and IL-1β (50% inhibition).^61^ These results align with previous studies on ovine digestates (<3 kDa), which exhibited ∼43% inhibition of IL-6 expression, while bovine digestates (<3 kDa) resulted in ∼21% inhibition.^12^

**Figure 5:**
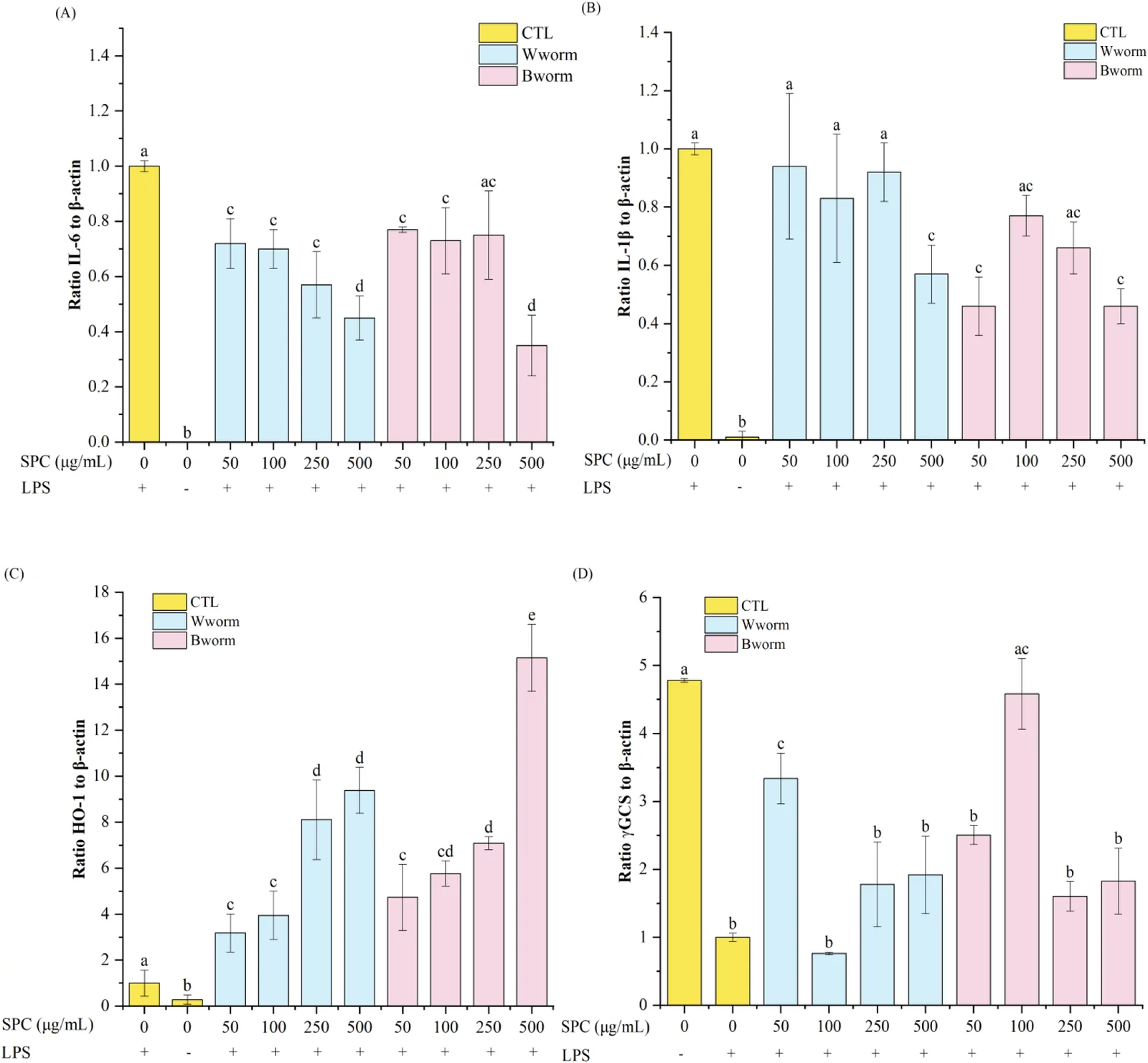
Dose-dependent effects of*Galleria mellonella*­(W) worm and*Alphitobius diaperinus*­(B) worm intestinal digestates (<10 kDa) with different soluble protein concentrations (SPC) on pro-inflammatory (**A**) IL-6 (**B**) IL-1β, and antioxidant target genes (**C**) HO-1 (**D**) γGCS in RAW264.7 macrophages. Data are the mean with SD of three independent experiments in triplicate. Different letters indicate significant differences, p < 0.05, Tukey test.

Several studies have shown that bioactive compounds can modulate the expression of antioxidant enzymes such as heme oxygenase-1 (HO-1) and γ-glutamylcysteine synthetase (γGCS), as target genes of the Nrf2 signaling pathway. Under normal conditions, Nrf2 is bound to Keap1 and targeted for degradation. Upon stimulation by bioactive compounds, Nrf2 is released, translocates to the nucleus, and binds to the antioxidant response element (ARE), thereby promoting the transcription of HO-1 and γGCS. HO1 degrades prooxidant heme into antioxidant metabolites, while γGCS, is the rate-limiting enzyme in glutathione synthesis, further enhances the cellular capacity for ROS detoxification. A recent study demonstrated that sugarcane-derived bioactive compounds enhanced stress resilience and longevity in *Caenorhabditis elegans* through transcriptomic mechanisms involving antioxidant and anti-inflammatory pathways.^63^ However, there is limited knowledge regarding the effects of insect protein-derived peptides on Nrf2 signaling and target gene expression. To determine whether the digestates altered the expression of genes associated with Nrf2-responsive antioxidant processes, HO-1 and γGCS mRNA abundance was quantified. Both digestates increased HO-1 and γGCS transcript levels relative to the LPS-treated control. These findings are consistent with modulation of Nrf2-responsive transcription but do not demonstrate activation of the canonical Nrf2 pathway. Nrf2 nuclear translocation, antioxidant response element activity, Keap1 abundance, and HO-1 and γGCS protein levels were not assessed. Therefore, the involvement of Nrf2 signaling remains a mechanistic hypothesis requiring direct protein-level and pathway-specific validation. For comparison, Song et al.^64^ also suggested that the HO-1 expression induced by sea cucumber peptides may be one of the possible mechanisms by which it exerts anti-inflammatory activity. Similarly, the antioxidant peptide purified from*Bacillus amyloliquefaciens* upregulated HO-1 expression by 2-fold.^65^ Within the nontoxic concentration range, W-I2h and B-I2h may provide a higher cellular protection. Regarding γGCS, the results suggest a threshold effect rather than a classical dose-dependent response. Several limitations should be considered when interpreting these findings. First, the anti-inflammatory effects were demonstrated using complex digestate fractions rather than purified or chemically synthesized peptides; therefore, the contribution of individual peptides remains to be confirmed. Second, molecular docking was used only as an exploratory tool and does not provide direct evidence of IKK-β inhibition or target engagement. Third, although changes in inflammatory cytokines and Nrf2-associated target gene expression were observed, protein-level confirmation of NF-κB/Nrf2 pathway modulation, including IκBα phosphorylation/degradation, p65 nuclear translocation, Nrf2 nuclear translocation, and HO-1 protein expression, was not performed. Fourth, the modified INFOGEST digestion excluded bile salts and lipolytic components, which may influence peptide release and bioavailability under physiological intestinal conditions. Finally, *in vivo* validation is required to assess peptide stability, absorption, safety, and functional relevance.

This study provides evidence that the <10 kDa intestinal-phase digestates generated from*Galleria mellonella*and *Alphitobius diaperinus*proteins, using a modified INFOGEST protocol, modulated inflammatory responses in LPS-stimulated macrophage models. Higher digestate concentrations reduced NF-κB reporter activity, nitric oxide production, and selected pro-inflammatory cytokine responses, whereas 25 μg soluble protein/mL increased IL-1β secretion, demonstrating a non-monotonic response. Increased HO-1 and γGCS transcript abundance was consistent with modulation of Nrf2-responsive gene expression, although pathway activation was not directly confirmed. LC-MS/MS identification, bioinformatic screening, and exploratory IKK-β docking prioritized GGSAASLGDHPHLGGLL as a candidate sequence for future validation. Direct testing of purified or synthetic peptides, confirmation of molecular target engagement, and assessment of stability, bioavailability, safety, and efficacy are required before peptide-level anti-inflammatory activity can be established.

## Abbreviations Used

W: *Galleria mellonella*
B: *Alphitobius diaperinus*
HO-1: heme oxygenase 1
IKKs: IκB kinases
Nrf2: nuclear factor erythroid 2-related factor 2
ROS: reactive oxygen species
NO: nitric oxide
SSF: salivary fluid
SGF: gastric phase fluid
SIF: intestinal phase fluids
DMEM: Dulbecco’s Modified Eagle Medium
LPS: lipopolysaccharide
W-I2h: Digestates from *Galleria mellonella*
B-I2h: Digestates from *Alphitobius diaperinus*
